# Androgen receptor expression and breast cancer mortality in a population-based prospective cohort

**DOI:** 10.1007/s10549-017-4343-0

**Published:** 2017-06-22

**Authors:** Karin Elebro, Pär-Ola Bendahl, Helena Jernström, Signe Borgquist

**Affiliations:** 1 Division of Oncology and Pathology, Clinical Sciences Lund, Lund University, Lund, Sweden; 2 Department of Plastic and Reconstructive Surgery, Skåne University Hospital, Jan Waldenströms gata 18, 205 02 Malmö, Sweden; 3 Clinical Trial Unit, Forum South, Skåne University Hospital, Lund, Sweden

**Keywords:** Breast cancer, Androgen receptor, Estrogen receptor, Prognostic marker, Breast cancer mortality

## Abstract

**Purpose:**

The increase in clinical trials with androgen receptor (AR)-targeting drugs emphasizes the need of clarifying the role of AR expression in different breast cancer subtypes. AR confers good prognosis in estrogen receptor positive (ER+) breast cancer, but its role in ER-negative (ER−) breast cancer is unclear. The aim of this study was to elaborate on previous findings of a differential prognostic role for AR depending on ER status, using breast cancer mortality (BCM) as endpoint, in a population-based cohort from the Malmö Diet and Cancer Study.

**Methods:**

Immunohistochemical AR expression was assessed in 910 women with invasive breast cancer diagnosed 1991–2010, supplemented with clinicopathological information, vital status, and cause of death, with the last follow-up in December 2014 (median 10 years). Survival analyses according to AR status and AR/ER combinations were performed.

**Results:**

AR expression was available for 671 tumors. AR+ (*n* = 573, 85%) was associated with favorable established tumor markers and lower BCM in univariable analysis, especially during the first 5 years following diagnosis [HR 0.4; 95% confidence intervals (CI) 0.2–0.7]. Multivariable analysis for short-term follow-up indicated higher BCM among patients with AR+ER− tumors (HR 3.5; 95% CI 1.4–9.1) than other AR and ER combinations.

**Conclusions:**

AR expression added prognostic information to ER expression with respect to short-term prognosis. The worst prognosis was seen for patients with AR+/ER− tumors in short-term follow-up, supporting the pre-specified hypothesis. However, larger cohorts are needed for further characterization of the role of AR expression in ER− breast cancer.

## Introduction

The heterogeneous nature of breast cancer highlights the importance of identifying prognostic and predictive markers for clinical implementation of tailored treatment. Recently, there has been increasing interest in the androgen receptor (AR) as a potential prognostic biomarker and treatment target [1, 2]. The prognostic role of AR has consistently been described as favorable for breast cancer in general and within the estrogen receptor-α positive (ER+) subgroup [3]. Some studies have reported that AR is prognostically beneficial irrespective of ER [4], but the results regarding the role of AR expression in the ER negative (ER−) setting diverge [2, 4–8]. Ongoing clinical trials are evaluating anti-androgens and selective androgen receptor modulators across different breast cancer subtypes [2, 9, 10]. It is essential to better define AR’s prognostic role in different breast cancer subtypes.

The most recent meta-analysis reported AR to be prognostically favorable irrespective of ER expression, but only included studies published until early 2015 [11]. In late 2015, we reported a differential role of AR depending on the ER status of the tumor using disease-free survival (DFS) as endpoint in a large population-based observational cohort. Patients with AR-positive (AR+) ER+ tumors had superior prognosis compared to all other AR/ER combinations. Markedly, the patients with AR+ER− tumors had worse prognosis in all adjusted models compared to patients with AR-negative (AR−) ER− tumors, and there was significant interaction between AR and ER expression with respect to DFS [8].

The aim of this study was to validate and elaborate the previous findings of a differential role for AR on the outcome depending on ER status, using breast cancer-related death as endpoint, in an independent cohort with longer follow-up, the population-based Malmö Diet and Cancer Study (MDCS). We hypothesized that AR positivity would be associated with overall favorable tumor characteristics and prognosis and that analyses stratified by tumor ER status would reveal a differential role of AR depending on ER. The primary endpoint was breast cancer mortality (BCM), and the secondary endpoint was all-cause mortality. Another aim was to investigate whether AR could add long-term prognostic information.

## Materials and methods

### The Malmö Diet and Cancer Study

The population-based prospective MDCS was initiated to examine associations between diet and cancer [12] and included people living in Malmö, Sweden in 1991–1996. Swedish language skills and mental abilities sufficient to understand the questionnaire were required for enrollment. The participation rate was 40% of the source population. A previous report showed lower mortality due to cancer and lifestyle-related causes among participants during recruitment and follow-up compared to non-participants [13]. Baseline data were collected from interviews, questionnaires, and examinations.

The female MDCS cohort consists of 17,035 women (born 1923–1950). Information on incident breast cancer and vital status were annually retrieved from the Swedish Cancer Registry, the South Swedish Regional Tumor Registry, and the Swedish Cause of Death Registry. Ethical permission was obtained from the Ethical Committee at Lund University (Dnr 472/2007). All participants signed a written informed consent form.

### Study population and patient characteristics

**Fig. 1:**
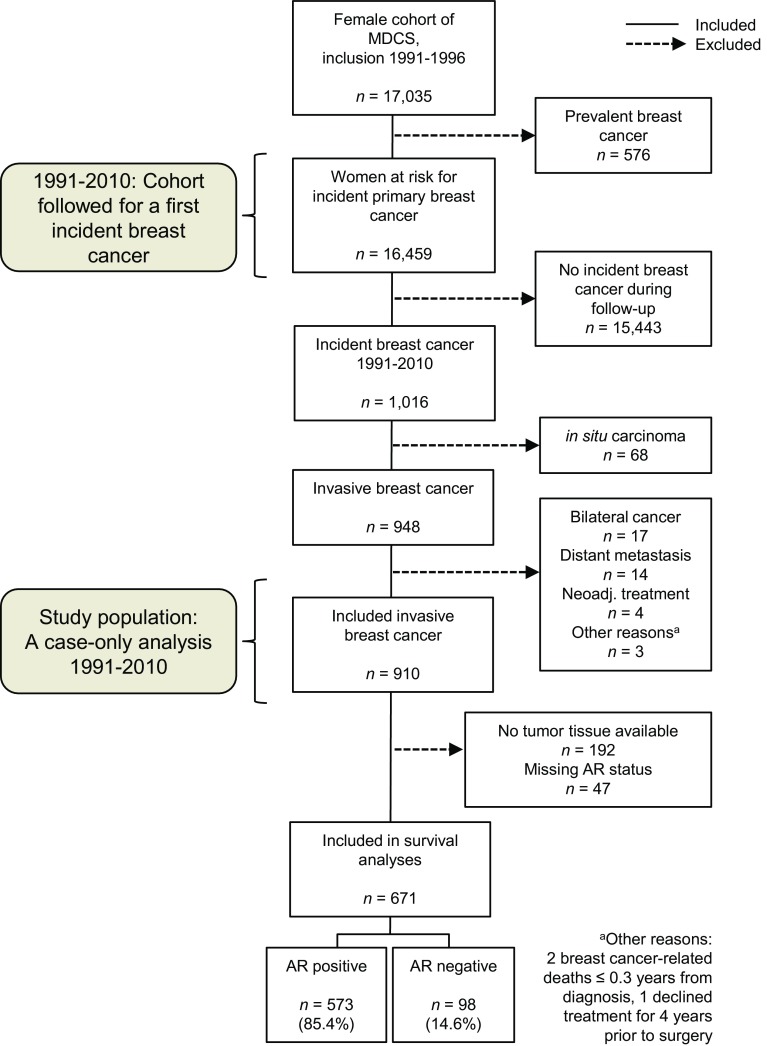
Flow chart of the Malmö Diet and Cancer Study (MDCS) and the study population

This is a case-only analysis of incident primary breast cancer within the MDCS, and patients with a previous breast cancer diagnosis at enrollment (*n* = 576) were therefore excluded (Fig. 1). During follow-up until December 31, 2010, a total of 1016 women were identified with incident breast cancer through record linkage. Patient characteristics at diagnosis were obtained from medical records. To investigate invasive tumor characteristics in relation to survival, in situ only cancers (*n* = 68) and patients who received neo-adjuvant treatments (*n* = 4) were excluded. Patients were also excluded if they had distant metastasis at diagnosis (*n* = 14) or died from breast cancer-related causes ≤0.3 years from diagnosis (*n* = 2). Patients with bilateral cancers (*n* = 17) were excluded due to difficulties in evaluating the relation between tumor characteristics and prognosis. Finally, one patient who declined treatment for four years before accepting surgery was excluded. Thus, the final study population consisted of 910 patients. Information on cause of death and vital status was retrieved from the Swedish Causes of Death Registry, with last follow-up December 31st, 2014.

### Tumor and histopathological analyses

Information on tumor size, grade, and axillary lymph node involvement (ALNI) was retrieved from pathology reports for tumors from 2005 and onwards. From 2008 onwards, information was also obtained for the immunohistochemistry (IHC) based markers ER, progesterone receptor (PR), proliferation index (Ki67), and human epidermal growth factor receptor-2 (HER2) status. From 1991 to 2007, ER, PR, Ki67 and HER2 were assessed using tissue microarrays (TMAs) [14]. A pathological re-evaluation was performed regarding invasiveness and grade for tumors diagnosed prior to 2006 [15]. A cut-off for positivity of >10% positively stained nuclei was applied for ER, PR, and Ki67. Regarding HER2, in situ hybridization (ISH) results were used when available. HER2 IHC was considered positive (HER2+) when annotated as 3+ and negative (HER2−) for 0 or 1+. HER2 IHC scorings of 2+ were categorized as missing if not confirmed to be amplified or normal in ISH analyses.

**Fig. 2:**
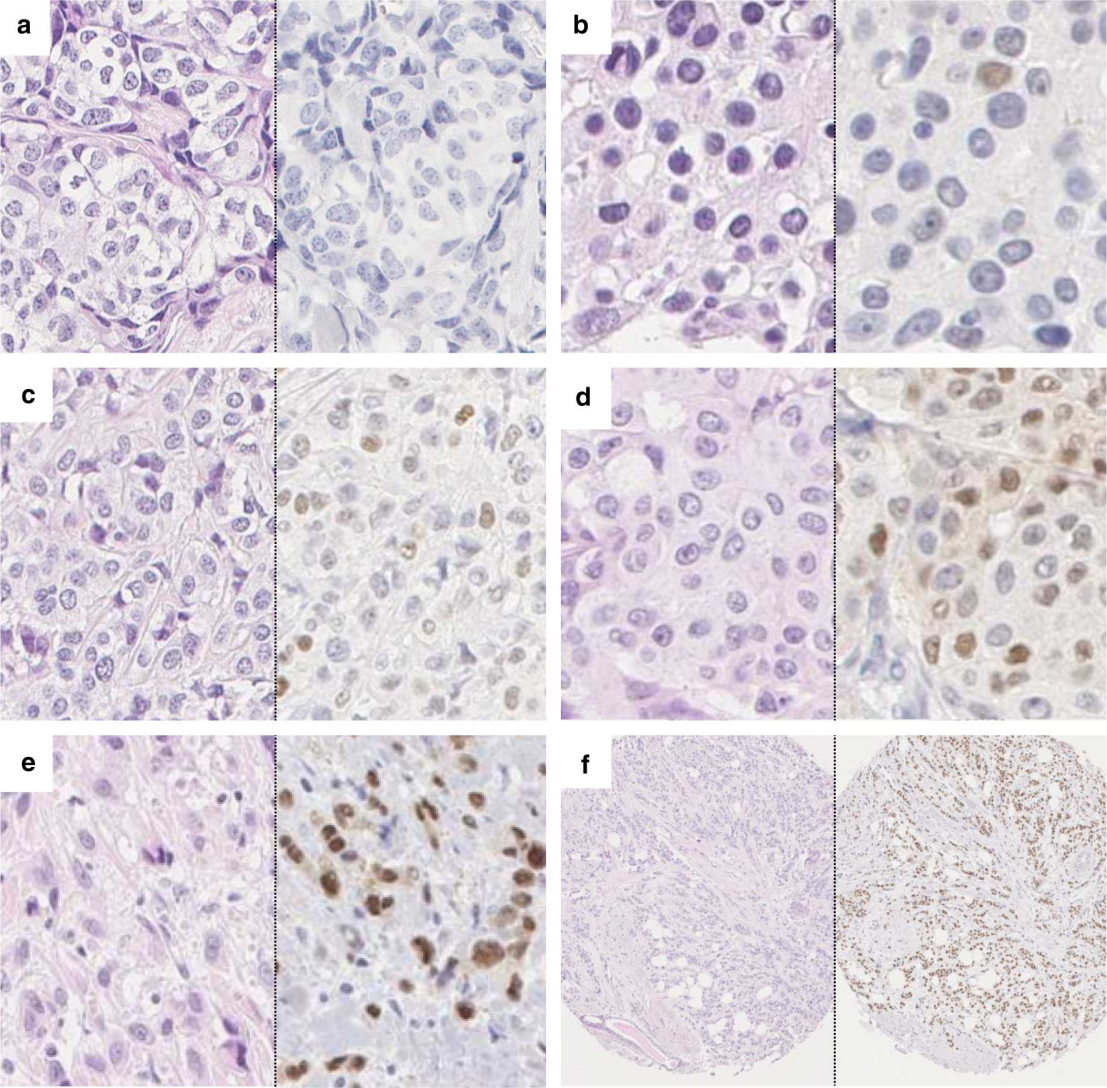
**a**–**f** Histology of invasive breast cancer, hematoxylin eosin stain (left) and immunohistochemistry of AR expression (right): **a** 0 % positively stained nuclei, **b** 1-10%, **c** 11-50%, **d** 51-75%, **e** >75 %, **f** overview, >75%

A TMA was constructed with two 1 mm cores from each tumor (Beecher, WI, USA). Among the 910 patients included in the study population, tumor tissue was available from 718 patients (Fig. 1). For IHC analysis of AR, 4-μm sections were automatically pretreated using the PT Link system and stained (Autostainer Plus, Dako, DK) for monoclonal antibody Ab-1 (clone AR441, dilution 1:200, Thermo Scientific). Microscopy assessments were performed using digital pathology (PathXL, http://www.pathxl.com). Following assessment of tumor invasiveness using hematoxylin and eosin stained slides, a semi-quantitative scoring of AR fractions (0, 1–10, 11–50, 51–75, and 76–100%) of positively stained nuclei, irrespective of nuclear staining intensity, was performed by one observer (KE), Fig. 2. If the cores from one tumor were discordant, a final score was evaluated across both cores. The AR variable was dichotomized using a cut-off of 10% as pre-specified in a statistical plan aimed at elaborating on our previous findings [8], and to avoid an exploratory study design approach. This study adheres to the REMARK criteria [16].

### Statistical analyses

The distribution of patient and tumor characteristics at diagnosis in relation to AR expression was categorized and presented as percentages. Continuous variables are presented as the median and interquartile range (IQR). Distributional differences were assessed by *X* ^2^-analyses, logistic regression, or Mann–Whitney *U* tests as appropriate.

The association between AR and prognosis was examined with BCM as the primary endpoint. BCM was defined as the incidence of breast cancer-related death (when breast cancer was the cause of death or the contributing cause of death). The secondary endpoint was all-cause mortality, i.e., death from any cause. Follow-up was calculated from the date of breast cancer diagnosis to the date of breast cancer-related death, date of death from another cause, date of emigration or the end of follow-up as of December 31, 2014.

Firstly, AR expression in relation to BCM was assessed by comparison of cumulative BCM (CBCM) for patients with AR+ and AR− tumors in the overall population and stratified by ER status. Rather than analyzing cause-specific survival, this method [17] takes competing risks into account, which was considered relevant due to the relatively long follow-up (median 10 years) and high median age at diagnosis of the patients (64.9 years). AR in relation to all-cause mortality was assessed by analyzing cumulative all-cause mortality (CM), both overall and stratified by ER status. Differences in CBCM and CM between subgroups were evaluated by the LogRank test. AR in relation to BCM was further investigated by cause-specific Cox proportional hazards analysis with follow-up censored when a death from another cause occurred. Effects are presented as the hazard ratios (HR) and 95% confidence intervals (CI) for the overall population, stratified by ER status, and for combinations of AR and ER status using the AR+ER+ subgroup as reference.

Adjustments were made in two multivariable models including the following covariates. Model 1 included age at diagnosis (continuous), tumor size >20 mm (yes/no), ALNI (≥1 yes/no), histological grade (III vs. I–II), and ER status (±). Model 2 included the covariates from Model 1 with the addition of planned adjuvant treatments, chemotherapy (yes/no), radiotherapy (yes/no), and endocrine treatment (yes/no). An interaction term between AR and ER was added to the Cox models to evaluate the strength of evidence against the null hypothesis of no interaction between these factors on outcome. Proportional hazards assumptions were evaluated visually by inspecting the log minus log survival curves and formally using Schoenfeld’s test. There was weak evidence for a non-proportional hazards effect on AR ± for the overall follow-up (*P* = 0.06). The follow-up time was subsequently divided into categories of 0–5 years and >5 years, where the proportional hazards assumption was better met, and survival analyses by AR status were repeated for 0–5 years and >5 years of follow-up.

All tests were two-sided, and *P* values should be interpreted as the level of evidence against each null hypothesis. Nominal *P* values without adjustments for multiple testing are presented. The user-contributed program stcompet.ado for the statistics package Stata version 14.1 (StataCorp LP, College Station, TX, USA) was used to estimate cause-specific cumulative mortality. Stata was also used to draw the cumulative mortality graphs and to test proportional hazards assumptions. All other statistical analyses were performed in SPSS 22.0 (IBM).

## Results

### AR expression, patient, and tumor characteristics

Tumor AR expression was assessable in 671 of 718 cases (93%) where tumor tissue was available (Fig. 1). The distribution of AR expression included 573 AR+ tumors (85%) and 98 AR− tumors (15%). AR+ status was associated with smaller tumor size, lower histological grade, ER/PR co-expression, and low proliferation index (Ki67 ≤ 10%). Within the ER−PR-negative (PR–) subset, 34% (15/44) of HER2– tumors (triple-negative breast cancer, TNBC) were AR+, and among HER2-positive tumors, 86% (12/14) were AR+. More patients with AR− tumors had received adjuvant chemotherapy and died from breast cancer-related causes compared to patients with AR+ tumors (Table 1).

**Table 1:** Distribution of patient and tumor characteristics

All *n* = 910						
Tumor in tissue microarray, *n* (%)		Yes, 718 (79%)				No, 192 (21%)
Androgen receptor (AR) expression assessable, *n* (%)		Yes, 671 (93%)			No, 47 (7%)	
AR negative (AR−) or AR positive (AR+), *n* (%)		AR− 98 (15%)	AR+ 573 (85%)		Missing AR	Missing tissue and AR
Factor	*n* (%) or median (IQR)	*n* (%) or median (IQR)	*n* (%) or median (IQR)	*P* ^a^	*n* (%) or median (IQR)	*n* (%) or median (IQR)
Age at baseline, years (*n* = 910)	55.4 (50.1–61.7)	55.1 (50.1–60.3)	54.9 (49.9–61.5)	0.94^b^	53.5 (48.4–60.8)	58.2 (51.7–63.3)
Age at diagnosis, years (*n* = 910)	65.0 (60.0–71.6)	64.0 (59.8–69.4)	65.0 (60.2–72.0)	0.22^b^	61.5 (55.7–70.1)	66.0 (60.4–71.9)
ER status (*n* = 760)						
Negative (≤10%)	89 (12)	37 (41)	39 (7)		5 (16)	8 (7)
Positive (>10%)	671 (88)	54 (59)	489 (93)	<0.0001	27 (84)	101 (93)
PR status (*n* = 689)						
Negative (≤10%)	311 (45)	58 (69)	190 (40)		17 (59)	46 (47)
Positive (>10%)	378 (55)	26 (31)	288 (60)	<0.0001	12 (41)	52 (53)
Tumor size (*n* = 887)						
>20 mm	250 (28)	47 (48)	160 (28)		9 (20)	34 (20)
≤20 mm	637 (72)	51 (52)	410 (72)	<0.0001	37 (80)	139 (80)
ALNI (*n* = 819)						
Positive (≥1 metastatic node)	262 (32)	39 (41)	182 (34)		9 (21)	32 (23)
Negative	557 (68)	55 (59)	361 (66)	0.13	34 (79)	107 (77)
NHG (*n* = 835)				<0.0001^c^		
I	227 (27)	9 (9)	158 (28)	Ref.^d^	13 (32)	47 (35)
II	392 (47)	30 (31)	285 (51)	0.12^d^	16 (40)	61 (46)
III	216 (26)	58 (60)	121 (21)	<0.0001^d^	11 (28)	26 (19)
HER2 status (*n* = 593)						
Positive (IHC/ISH)	52 (9)	6 (9)	39 (9)		2 (7)	5 (6)
Negative	541 (91)	64 (91)	372 (91)	0.81	28 (93)	77 (94)
In ER negative PR negative only (*n* = 79)						
HER2 status (*n* = 67)						
Positive	18 (27)	2 (6)	12 (44)		1 (25)	3 (60)
Negative (TNBC)	49 (73)	29 (94)	15 (56)	0.0007	3 (75)	2 (40)
Ki67 status (*n* = 633)						
High (>10%)	214 (34)	43 (55)	131 (30)		13 (48)	27 (31)
Low (≤10%)	419 (66)	35 (45)	309 (70)	<0.0001	14 (52)	61 (69)
Planned adjuvant treatments						
Endocrine therapy						
In all patients (*n* = 878)						
None	409 (47)	54 (56)	213 (38)		29 (62)	113 (66)
Any (TAM/AI)	469 (53)	43 (44)	349 (62)	0.001	18 (38)	59 (34)
In ER+ only (*n* = 662)						
None	254 (38)	17 (31)	166 (35)		13 (48)	58 (58)
Any (TAM/AI)	408 (62)	37 (69)	315 (65)	0.66	14 (52)	42 (42)
Chemotherapy (*n* = 823)						
Yes	126 (15)	39 (40)	67 (13)		5 (11)	15 (9)
No	697 (85)	58 (60)	448 (87)	<0.0001	39 (89)	152 (91)
Radiotherapy (*n* = 825)						
Yes	499 (60)	60 (63)	328 (63)		23 (52)	88 (53)
No	326 (40)	35 (37)	191 (37)	0.99	21 (48)	79 (47)
Death during follow-up (*n* = 256)	256	34	144		16	62
Breast cancer underlying cause	109 (43)	23 (68)	55 (38)	<0.0001	4 (25)	27 (43)
Breast cancer contributing cause	23 (9)	1 (3)	14 (10)	0.38	2 (12)	6 (10)
Other causes	124 (48)	10 (29)	75 (52)	0.43	10 (63)	29 (47)

*AI* aromatase inhibitors, *ALNI* axillary lymph node involvement, *AR* androgen receptor, *CI* confidence interval, *df* degree of freedom, *ER* estrogen receptor alpha, *HER2* human epidermal growth factor 2 *HR* hazard ratio, *IHC* immunohistochemistry, *IQR* interquartile range, *ISH* in situ hybridization, *Ki67* proliferation index, *NHG* Nottingham Histological Grade, *PR* progesterone receptor, *TAM* tamoxifen, *TNBC* triple-negative breast cancer

^a^ *Χ* ^2^ test if not specified otherwise

^b^Mann–Whitney *U* test

^c^ *Χ* ^*2*^ 2df

^d^Logistic regression model, grade I as reference

### Cumulative breast cancer mortality and all-cause mortality by AR expression

**Fig. 3:**
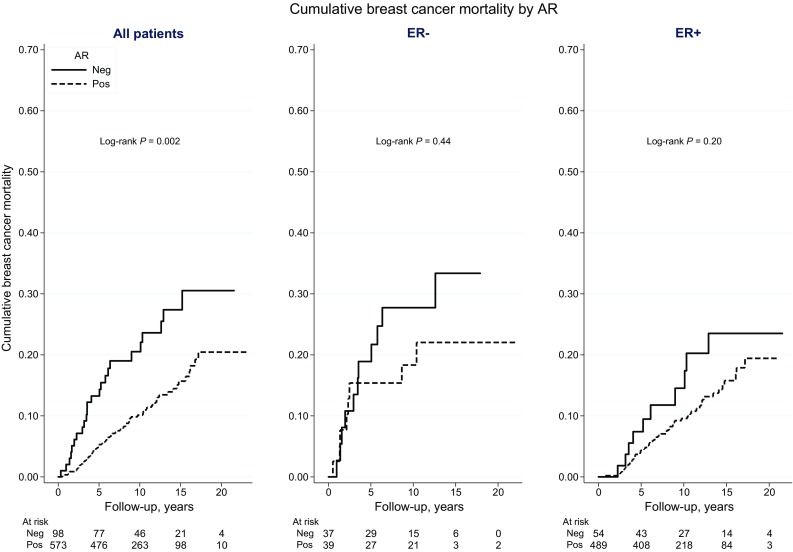
Cumulative breast cancer mortality according to AR status, among all patients and stratified by ER status

**Fig. 4:**
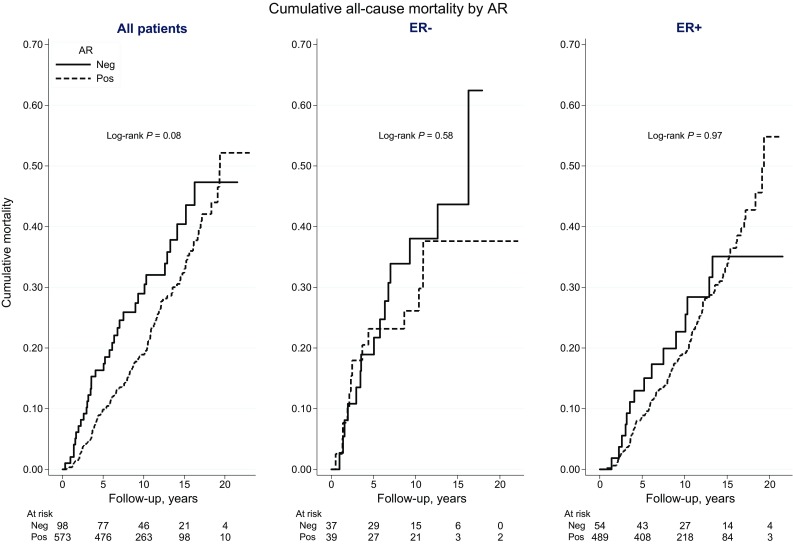
Cumulative all-cause mortality according to AR status, among all patients and stratified by ER status

During follow-up, 178 of the patients included in survival analyses died, of whom 93 died from breast cancer-related causes (Table 1). The median follow-up period for patients who were still alive by the end of 2014 was 10 years. The incidence of breast cancer-related death was graphically illustrated by CBCM plots (Fig. 3). CBCM was lower among patients with AR+ tumors compared to patients with AR− tumors (LogRank *P* = 0.002). When stratified by ER status, the CBCM observed among all patients with AR+ tumors was attributable to patients with AR+ER+ rather than AR+ER− tumors. However, the evidence for an association between AR+ER+ and lower CBCM was weak (LogRank *P* = 0.20) compared to AR−ER+. During the first 5 years of follow-up, no difference in CBCM by AR+ was observed for patients with ER− tumors. AR+ was weakly associated with higher all-cause mortality (LogRank *P* = 0.08), but this association was not seen in CM analyses stratified by ER status (Fig. 4). Cox regression analyses were performed to further investigate CBCM in relation to AR status, as well as to ER status and time from diagnosis to breast cancer-related death. No significant prognostic differences depending on diagnostic period before and after the trastuzumab introduction (≤year 2005 vs. 2006+) was revealed.

### Breast cancer mortality by AR and ER expression for overall follow-up

For the overall follow-up period, the incidence of BCM for patients with AR+ tumors was half that of patients with AR− tumors in univariable Cox analyses (HR 0.48: 95% CI 0.30–0.77; *P* = 0.002, Table 2). However, after adjustments for potential confounders, no risk reduction by AR+ remained (Table 2, models 1 and 2). When associations between AR and incidence of breast cancer-related death were assessed in the ER+ and ER− subgroups separately, no prognostic impact by AR was seen in either group. Furthermore, no interaction was observed between AR and ER (*P*_interaction_ ≥ 0.58) regarding BCM.

**Table 2:** Breast cancer mortality according to AR status, stratified by ER status and according to combinations of AR and ER status. Crude and adjusted models are presented for the overall follow-up period, for 0–5 years and >5 years of follow-up

Follow-up period and subgroup			HRs								
Follow-up			Crude			Model 1^a^			Model 2^b^		
Subgroups	Total (n)	Events (n)	HR	95% CI	*P*	HR	95% CI	*P*	HR	95% CI	*P*
Overall											
AR+ vs. AR−											
All patients	671	93	0.48	0.30–0.77	0.002	1.06	0.59–1.92	*0.84*	1.12	0.62–2.04	*0.71*
ER+	543	65	0.64	0.33–1.26	0.20	0.95	0.47–1.92	*0.89*	1.02	0.51–2.04	*0.97*
ER−	76	19	0.70	0.28–1.75	0.45	1.44	0.46–4.54	*0.53*	1.16	0.34–4.00	*0.81*
					0.91^c^			*0.58* ^c^			*0.63* ^c^
ER missing^d^	52	9	0.17	0.04–0.76	0.02	–^e^	–	–	–^e^	–	–
AR and ER											
ER+AR+	489	55	Ref.			Ref.			Ref.		
ER−AR−	37	11	2.94	1.54–5.62	0.001	1.49	0.73–3.03	*0.27*	0.83	0.33–2.07	*0.69*
ER+AR−	54	10	1.57	0.80–3.08	0.19	1.05	0.53–2.11	*0.88*	0.98	0.49–1.96	*0.95*
ER−AR+	39	8	2.01	0.95–4.22	0.07	1.96	0.91–4.20	*0.08*	1.14	0.47–2.77	*0.78*
0–5 years											
AR+ vs. AR−											
All patients	671	43	0.37	0.19–0.71	0.003	1.33	0.56–3.15	0.52	1.22	0.51–2.92	0.66
ER+	543	25	0.56	0.19–1.62	0.28	0.91	0.30–2.73	0.86	0.93	0.31–2.81	0.89
ER−	76	13	0.84	0.28–2.49	0.75	1.73	0.51–5.92	0.38	1.37	0.38–4.97	0.63
					0.60^c^			0.35^c^			0.50^c^
ER missing	52	5	0.20	0.03–1.19	0.08	–^e^	–	–	–^e^	–	–
AR and ER											
ER+AR+	489	21	Ref.			Ref.			Ref.		
ER−AR−	37	7	4.85	2.06–11.41	0.0003	1.86	0.74–4.67	0.18	1.36	0.38–4.82	0.63
ER+AR−	54	4	1.78	0.61–5.19	0.29	1.10	0.37–3.29	0.87	1.06	0.35–3.20	0.91
ER−AR+	39	6	4.09	1.65–10.14	0.002	3.55	1.38–9.13	0.009	2.23	0.68–7.27	0.19
>5 years											
AR+ vs. AR−											
All patients	553	50	0.61	0.31–1.20	0.15	0.91	0.41–2.02	0.83	1.09	0.48–2.45	0.83
ER+	451	40	0.70	0.29–1.68	0.43	0.98	0.40–2.41	0.96	1.08	0.44–2.66	0.87
ER−	56	6	0.46	0.08–2.57	0.38	0.12	0.001–12.36	0.37	–^e^	–	–
					0.65^c^			–^e^			–^e^
ER missing	41	4	0.11	0.007–1.82	0.12	–^e^	–	–	–^e^	–	–
AR and ER											
ER+AR+	408	34	Ref.			Ref.			–		
ER−AR−	29	4	1.75	0.62–4.92	*0.29*	1.17	0.37–3.68	0.78	–^e^	–	–
ER+AR−	43	6	1.43	0.60–3.42	*0.42*	1.02	0.42–2.52	0.96	–	–	–
ER−AR+	27	2	0.78	0.19–3.27	*0.74*	0.83	0.20–3.53	0.80	–	–	–

*AR* androgen receptor, *CI* confidence interval, *ER* estrogen receptor alpha, *HR* hazard ratio

^a^Adjusted for age at diagnosis (continuous), tumor size >20 mm yes/no, ALNI ≥ 1 metastatic node yes/no, Grade III yes/no and ER status ± . Complete case analysis. 80 patients excluded in the analysis of all patients due to missing values for one or more of the variables in the model

^b^Adjusted as in model 1, but also for planned adjuvant treatments (chemotherapy yes/no, radiotherapy yes/no, endocrine treatment yes/no). Complete case analysis. 138 patients excluded in the analysis of all patients due to missing values for one or more of the variables in the model

^c^ *P* value for interaction

^d^AR distribution among ER missing: 45 AR+ and 7 AR−

^e^Too few patients left for meaningful analyses

In the analyses of AR/ER combinations, patients with AR−ER− as well as AR+ER− tumors had increased risk of CBCM compared to patients with AR+ER+ tumors. After adjustments for age and tumor characteristics (Table 2, model 1), there was slight evidence that patients with AR+ER− tumors had higher incidence of breast cancer-related death compared to patients with AR+ER+ tumors. However, this association did not remain after adjusting for adjuvant treatments.

### Breast cancer mortality by AR and ER in short-term versus long-term follow-up

Separate analyses were performed for the initial 5-year period and from 5 years onwards (Table 2). This was done to better meet the assumption of proportional hazards and to differentiate between potential early and late impacts of tumor AR on BCM.

The lower BCM in the AR+ group observed in the overall period seemed driven by a lower BCM relative to AR− during the first 5 years (HR 0.37: 95% CI 0.19–0.71; *P* = 0.003, 43 events). The corresponding figures for the interval from five years and onwards pointed in a similar direction, although non-significant (HR 0.61: 95% CI 0.31–1.20; *P* = 0.15, 50 events). In the adjusted models for ER+ versus ER− strata during the short-term follow-up, the estimated hazard ratios for BCM by AR diverged to a higher degree compared to the corresponding estimate in the overall follow-up period. However, no interactions between AR and ER status were observed (*P*_interaction_ ≥ 0.35). After adjustments for age and tumor characteristics (model 1, 0–5 years), there was moderate evidence that patients with AR+ER− tumors had higher BCM during the short-term follow-up compared to patients with AR+ER+ tumors. In this model, BCM for patients with AR+ER− tumors was more than threefold that of patients with AR+ER+ tumors. Furthermore, patients with AR+ER− tumors had almost double the BCM compared with patients with AR−ER− tumors. When further adjustments for adjuvant treatment were incorporated into the model (model 2), the patients with AR+ER− tumors still had worse prognosis compared to all other AR/ER combinations.

## Discussion

In this prospective cohort with long-term follow-up, breast cancer patients with AR+ tumors had lower BCM but not all-cause mortality compared to patients with AR− tumors. AR+ was not an independent prognostic factor, and the hypothesis of an interaction between AR and ER was not confirmed. However, in line with our hypothesis, patients with AR+ER− tumors had the worst prognosis of all AR/ER combinations, as demonstrated in multivariable analyses with short-term follow-up.

The current interest in AR in breast cancer is multilayered. AR is a promising primary target for treatment in ER− breast cancer and especially in TNBC, where treatment options are scarce. Furthermore, specific molecular subgroups with androgen-regulated genetic signatures have emerged [18–20]. For example, the Luminal AR (LAR) subtype has been associated with a clearly different clinical course from that of the other TNBC subtypes [21–23].

In ER+ disease, AR antagonizes the proliferative effect of ER signaling [24]. AR may improve prediction of endocrine response [25, 26] and also offer an alternative endocrine treatment target when anti-estrogen regimens fail. Studies focused on integrating AR with other transcription factors such as ER are warranted to better understand the associations between AR and survival across breast cancer subtypes [2]. One possible next step would be to address AR as a ratio of ER, as done previously [22]. This may enhance the understanding of AR actions, due to the suggested competitive interactions between these two receptors [27].

We conclude that AR adds information for BCM compared to that of ER alone, but in the present study only to a limited extent. The findings of AR status on BCM did not remain after adjustments for confounders, suggesting that AR did not drive the association. Instead, ER was interpreted as the primary driver since AR and ER were highly co-expressed, and the association with BCM remained for patients with ER+ tumors in short-term adjusted analyses (data not shown). This interpretation was supported by the unadjusted analysis of combined AR/ER. However, in line with our hypothesis, AR added short-term prognostic information after adjustment for confounders. The poorest prognosis was seen among patients with AR+ER− tumors. This was also true after adjustment for treatment, but the adjusted HRs were closer to 1.0, and the evidence for higher BCM compared to the other subgroups was weaker after adjustment. If AR+ER− breast cancer is confirmed to have inferior prognosis, this may impact the choice of AR-targeted treatments and reveal a need for closer surveillance of this patient group.

There are several suggested reasons as to why we could not fully confirm our hypothesis. Our previous results on AR expression considered DFS in a cohort of median follow-up of 5 years. The time from diagnosis to breast cancer-related death may not be comparable with the event of local or regional recurrence, contralateral cancer, or distant metastasis, as evaluated previously [8]. There may also be biological differences in the AR effect in relation to these different endpoints. For the follow-up beyond 5 years, there was a selection of healthier patients with more favorable biology. Furthermore, the patients in the MDCS were diagnosed earlier (from 1991 to 2010) and during a 19-year period, with indications for treatment regimens such as tamoxifen treatment changing over time, where tamoxifen was initially prescribed according to menopausal status rather than ER status [28]. Our initial results [8] were based on the BC Blood Study, which was initiated in 2002 and included more aromatase inhibitor-treated patients.

AR+ was confirmed to be associated with favorable tumor characteristics, as consistently shown previously [8, 29, 30]. In the present study, the proportion of patients with AR+ tumors (85%) was equal to that of the independent cohort BC Blood Study (AR+ 85%), in which the same antibody was used [8]. This provides a measure of external validity to the AR assessments, since similar exclusion criteria were adopted. Both studies involved population-based Swedish cohorts, and similar distributions could thus be expected.

The strengths in this study were the long-term follow-up and a study population from a population-based, well-characterized cohort [13, 31]. Since the median age at diagnosis was greater than 60 years, the findings are mostly applicable to postmenopausal breast cancer. The completeness of the Swedish Cancer Registry is considered to be high with low underreporting of breast cancer [32]. The Swedish Cause of Death Registry has been reported to have complete and valid data from an international perspective, with the highest accuracy for cancer diagnoses [33].

A lack of standardization of AR antibodies and cut-offs used has been common and may explain some discrepancies across studies [4, 11]. In the present study, a validated antibody and a common cut-off were used [4]. The amount of missing AR values among patients for which tumor tissue was available was low and was similar in distribution to the overall population and was not considered a to exert a risk for selection bias. However, patients with missing tumor tissue were older and had smaller tumors, with a lower proliferation index and grade. They also more often had node-negative disease, suggesting under-representation of tumors with favorable characteristics in the material, which constitutes a selection bias. Thus, the true proportion of AR+ may be slightly higher than the value presented in this paper, and the incidence of BCM may be slightly lower in the underlying population from which the study population was sampled.

The tumor marker data were derived from TMAs for patients diagnosed up until 2007. Thereafter, routine clinical pathology reports were used. For the PR variable, this introduced a systematic bias due to higher positive frequency in clinical data as compared to TMA data. Thus, PR was only addressed in the descriptive analyses and should be interpreted with caution. In contrast, ER status did not depend on the diagnostic period. The missing ER was addressed by presenting the associated HRs separately.

This study indicates that cohorts with larger sample sizes are needed to further elucidate the prognostic role of AR in ER− breast cancer. Indeed, there is a need to incorporate several hormonal receptors in future studies of prognosis and treatment prediction [34]. AR may be valuable for short-term prognosis, whereas additional specific markers may be needed for long-term prognosis for ER+ breast cancer, which tends to relapse late [35].

The number of ongoing clinical studies involving AR targeting is steadily expanding and has included patients with both early and advanced breast cancer and of different subtypes [36–39] (ClinicalTrials.govidentifier: NCT01842321, NCT01889238, NCT02689427, NCT02676986, NCT02457910, NCT02463032, NCT02368691). Identification of subtypes based on gene expression analyses have provided a more comprehensive picture of hormonal regulation, such as the LAR subtype which is heavily enriched in hormonally regulated pathways in spite of being ER− [21].

There is yet no consensus on whether AR antagonists or agonists are preferable as targeted treatments in any breast cancer subtype. The forthcoming reports of ongoing clinical trials—combined with observational studies and mechanistic studies on treatment response/resistance [40–44]—will impact the future direction. Most likely, a wider clinical implementation of AR assessments will be of highest value for patients with TNBC [23] and for patients in the metastatic setting that have suffered from treatment resistance, and may benefit from the addition of AR-targeted treatments such as enzalutamide [22, 44–46].

In conclusion, AR+ was associated with lower BCM in the overall study population and for the overall and short-term follow-up intervals, but not for long-term follow-up. The poorest prognosis was seen among patients with AR+ER− tumors after adjustment for confounders in the short-term follow-up. Future studies on the role of AR in breast cancer require larger cohorts, especially in the ER− subset, and the inclusion of gene expression analyses may add valuable information.

## Abbreviations

ALNI: Axillary lymph node involvement
AR: Androgen receptor
BCM: Breast cancer mortality
CBCM: Cumulative breast cancer mortality
CI: Confidence interval
CM: Cumulative all-cause mortality
DFS: Disease-free survival
ER: Estrogen receptor alpha
HER2: Human epidermal growth factor receptor -2
HR: Hazard ratio
IHC: Immunohistochemistry
IQR: Interquartile range
ISH: In situ hybridization
Ki67: Proliferation associated antigen
LAR: Luminal AR
MDCS: Malmö Diet and Cancer Study
PR: Progesterone receptor
TMA: Tissue microarray
TNBC: Triple-negative breast cancer
